# Selective enhancement of the tumour necrotic activity of TNF alpha with monoclonal antibody.

**DOI:** 10.1038/bjc.1992.180

**Published:** 1992-06

**Authors:** D. A. Rathjen, L. J. Furphy, R. Aston

**Affiliations:** Peptide Technology Ltd., Sydney, NSW, Australia.

## Abstract

The binding and biological activity of human TNF alpha on endothelial and tumour cells has been studied in the presence of monoclonal antibodies (MAbs). In particular, one monoclonal antibody to TNF alpha (MAb 32) has been identified which failed to inhibit binding and cytotoxicity of TNF alpha on WEHI-164 tumour cells but which was a potent inhibitor of TNF alpha-induced endothelial cell procoagulant activity on bovine aortic endothelial cells. The ability of MAb 32 to inhibit selectively the actions of TNF alpha on endothelial cells but not on tumour cells suggests a mechanism for enhancement of the anti-tumour action of TNF alpha in vivo when in complex with this antibody. Treatment of tumour bearing mice (WEHI-164 and Meth A fibrosarcoma) with TNF alpha-MAb 32 complex resulted in a 5- to 10-fold enhancement in the potency of the cytokine in comparison to free TNF alpha. Complexes between this cytokine and other MAbs generally resulted in either no effect or inhibition of TNF alpha activity in vivo and in vitro. Neither intact MAb 32 nor FAb' fragments of MAb 32 showed any tumour regressive activity in the absence of TNF alpha. The FAb' fragments were equipotent to the bivalent form of the antibody in enhancing TNF alpha activity. These data provide evidence that it is possible to segregate the individual biological activities of TNF alpha with concomitant enhancement of the tumour regressive activity of the cytokine in vivo.


Br. J. Cancer (1992), 65, 852-856 © Macmillan Press Ltd., 1992

### Selective enhancement of the tumour necrotic activity of $TNF_\alpha$ with monoclonal antibody

D.A. Rathjen, L.J. Furphy & R. Aston

*Peptide Technology Ltd., 4–10 Inman Road, PO Box 444, Dee Why, Sydney, NSW 2099, Australia.*

**Summary** The binding and biological activity of human $TNF_\alpha$ on endothelial and tumour cells has been studied in the presence of monoclonal antibodies (MAbs). In particular, one monoclonal antibody to $TNF_\alpha$ (MAb 32) has been identified which failed to inhibit binding and cytotoxicity of $TNF_\alpha$ on WEHI-164 tumour cells but which was a potent inhibitor of $TNF_\alpha$-induced endothelial cell procoagulant activity on bovine aortic endothelial cells. The ability of MAb 32 to inhibit selectively the actions of $TNF_\alpha$ on endothelial cells but not on tumour cells suggests a mechanism for enhancement of the anti-tumour action of $TNF_\alpha$ *in vivo* when in complex with this antibody. Treatment of tumour bearing mice (WEHI-164 and Meth A fibrosarcoma) with $TNF_\alpha$-MAb 32 complex resulted in a 5- to 10-fold enhancement in the potency of the cytokine in comparison to free $TNF_\alpha$. Complexes between this cytokine and other MAbs generally resulted in either no effect or inhibition of $TNF_\alpha$ activity *in vivo* and *in vitro*. Neither intact MAb 32 nor FAb′ fragments of MAb 32 showed any tumour regressive activity in the absence of $TNF_\alpha$. The FAb′ fragments were equipotent to the bivalent form of the antibody in enhancing $TNF_\alpha$ activity. These data provide evidence that it is possible to segregate the individual biological activities of $TNF_\alpha$ with concomitant enhancement of the tumour regressive activity of the cytokine *in vivo*.

Tumour necrosis factor ($TNF_\alpha$) is a product of activated macrophages in response to infection and during malignancy. Systemic administration of this cytokine results in haemorrhagic necrosis of tumours *in vivo* (Carswell *et al.*, 1975; Green *et al.*, 1977) whereas *in vitro*, it has cytostatic and cytolytic activity on tumour cells (Helson *et al.*, 1975). In addition to its 'host-protective' effects, $TNF_\alpha$ has been implicated as the causative agent in the pathology associated with septicemia, cachexia, cerebral malaria and cancer. Although recombinant $TNF_\alpha$ has been used therapeutically in cancer patients, side-effects such as coagulopathy, thrombocytopaenia, lymphcytopaenia, hepatotoxicity and renal impairment have limited its application (Creaven *et al.*, 1987; Kimura *et al.*, 1987; Selby *et al.*, 1987; Naworth & Stern, 1986). The systemic toxicity associated with the administration of $TNF_\alpha$ is believed to be, at least in part, a consequence of its interaction with the endothelium (Bevilacqua *et al.*, 1986; Nawroth & Stern, 1986; Selby *et al.*, 1987). Furthermore, reducing the ability of $TNF_\alpha$ to bind to endothelial cells whilst preserving its tumour cytotoxic activity may have a beneficial outcome in the use of this cytokine therapeutically. We describe here a monoclonal antibody to human $TNF_\alpha$ which significantly enhances the tumour regression activity of the cytokine (5–10-fold) whilst inhibiting some of the associated toxic side-effects. In particular, the antibody has been shown to inhibit the procoagulant activity of $TNF_\alpha$ on endothelial cells whilst having no effect on the binding of the cytokine to WEHI-164 tumour cells. These observations may provide the basis for an improved approach to therapy with this cytokine.

### Materials and methods

### *Animals and tumour cell lines*

All experiments were performed using female BALB/c mice aged 10–12 weeks obtained from the CSIRO Division of Biomolecular Engineering animal facility. The WEHI-164 fibrosarcoma line was obtained from Dr Geeta Chauhdri (John Curtin School of Medical Research, Australian National University). The Meth A sarcoma lines were obtained from Dr Elizabeth Richards (Sloan Kettering Cancer Centre).

### *Fusions and production of monoclonal antibodies*

A panel of 13 murine monoclonal antibodies (MAbs) against human recombinant TNF was raised and characterised as previously described (Rathjen *et al.*, 1991). Two monoclonal antibodies are described here; one is a potent inhibitor of all the activities of $TNF_\alpha$ (MAb 47) and a second unique antibody (MAb 32) which selectively inhibits the effects of $TNF_\alpha$ on endothelial cells (see text).

### *WEHI-164 cytotoxicity assay*

Bioassay of recombinant $TNF_\alpha$ activity was performed according to the method described by Espevik and Nissen-Meyer (1986). Briefly, WEHI-164 cells were cultured in RPMI-1640 supplemented with 10% foetal calf serum, 10 mM hepes and penicillin-streptomycin. Prior to use in the assay of TNF activity the cells were harvested, washed in culture medium once and placed in wells of a 96 well microtray ($2 \times 10^4$ cells/well). $TNF_\alpha$ (Bissendorf Biochemicals $3.2 \times 10^7$ units $mg^{-1}$) at varying concentrations was then added to each well. Actinomycin D (5 $\mu g\ ml^{-1}$) was used to enhance the cytotoxic action of $TNF_\alpha$. Monoclonal antibodies were used at 1 $\mu g$ well. After 20 h in the presence of TNF and/or MAb MTT (3-(4,5-Dimethylthiazol-2yl)-2,5-diphenyltetrazolium bromide, 10 $\mu l$ of 5 $mg\ ml^{-1}$ stock) was added to each well. The cultures were incubated for a further 4 h before the supernatant was carefully removed and the insoluble precipitate dissolved by the addition of 100 $\mu l$ of acidified propan-2-ol to each well. The optical density was then read at 570 nm with a reference wavelength of 630 nm.

### *Tumour regression experiments*

Subcutaneous tumours were induced by the injection of approximately $5 \times 10^5$ cells (WEHI-164 or Meth A fibrosarcoma). This produced tumours of diameters of 10 to 15 mm approximately 14 days later at which time experiments commenced. Mice were injected i.p. for four consecutive days with recombinant human $TNF_\alpha$ (0.1 $\mu g$–10 $\mu g$) and MAb (50 $\mu g$ as ascitic globulin fraction prepared by sodium sulphate precipitation) mixed 60 min prior to administration.

Correspondence: D.A. Rathjen.
Received 23 September 1991; and in revised form 21 February 1992.

Control groups received injections of PBS alone, MAb alone or control MAb (MAb against bovine growth hormone) with $TNF_{\alpha}$. Tumour size was measured daily throughout the course of the experiment. Statistical significance of the results was determined by unpaired *t*-test.

*Radioreceptor assays*

WEHI-164 cells grown to confluency were scrape harvested and washed once with 1% bovine serum albumin in Hanks balanced salt solution (HBSS, Gibco) and used at $2 \times 10^6$ cells per assay sample. Bovine aortic endothelial cells (passage 6) were seeded ($4 \times 10^4$ cells per well) into 24 well culture dishes and grown to confluency (3–4 days) in McCoys 5A medium supplemented with 20% FCS, L-glutamine and penicillin/streptomycin (growth medium). For the radioreceptor assay, the cells were washed once in growth medium and then incubated with varying amounts of either unlabelled $TNF_{\alpha}$ ($1-10^4$ ng per assay sample) or MAb (10-fold dilutions commencing 1/10 to $1/10^5$ of ascitic globulin) and $^{125}$I-TNF (50,000 c.p.m.) labelled using the lactoperoxidase method as previously described (Aston *et al.*, 1985) for 3 h at 37°C in a shaking water bath (WEHI-164 cells) or 1 h at 37°C in a humidified $CO_2$ incubator (endothelial cells). At the completion of the incubation 1 ml of HBSS/BSA was added to the WEHI-164 cells, the cells spun and the bound $^{125}$I in the cell pellet counted. For the endothelial cell assay, 1 ml of growth medium was added to each well and aspirated followed by the addition of 0.1 ml of 0.1 M sodium hydroxide to lyse the cells. The cell lysate was then transferred to tubes for counting of bound $^{125}$I-TNF. Binding that could not be displace by an excess (1 µg) of unlabelled TNF was considered to be non-specific. Specific binding was calculated from total binding minus non-specific binding of triplicate assay tubes. One hundred per cent specific binding corresponded to 1,500 c.p.m.

*Endothelial cell clotting assays*

Endothelial cell procoagulant activity (PCA) induction by $TNF_{\alpha}$ was determined using bovine aortic endothelial cells (BAE) according to the procedure of Bevilacqua *et al.* (1986) with the following modifications: BAE cells were propagated in McCoys 5A medium supplemented with 10% FCS, penicillin, streptomycin and L-gutamine in standard tissue culture flasks and 24-well dishes. $TNF_{\alpha}$ treatment of cultures (3 µg $ml^{-1}$) was for 4 h at 37°C in the presence of growth medium after which the cells were washed and scrape-harvested before being frozen, thawed and sonicated. Total cellular PCA was determined in a standard one-stage clotting assay using normal donor platelet poor plasma to which 100 µl of $CaCl_2$ and 100 µl of cell lysate was added. The time taken for clotting to occur was then measured. Statistical significance was determined by unpaired *t*-test.

*Preparation of FAb' monoclonal antibody fragments*

Univalent antibody fragments were prepared by digestion of MAb 32 with agarose immobilised papain (Pierce) according to the manufacturer's instructions. FAb' and Fc antibody fragments were separated by protein A-Sepharose affinity chromatography (Pharmacia) and tested for size and binding to $^{125}$I-$TNF_{\alpha}$ by electrophoresis and radioimmunoassay respectively.

### Results

*Binding of $TNF_{\alpha}$-MAb complexes to tumour and endothelial cells*

The binding of $TNF_{\alpha}$ to cultured WEHI-164 tumour cells and bovine endothelial cells in the presence of anti-$TNF_{\alpha}$ MAbs is shown in Figure 1. Unlike MAb 47, which was found to inhibit $TNF_{\alpha}$ binding to both cell types, MAb 32 only inhibited $^{125}$I-$TNF_{\alpha}$ binding to endothelial cells. This correlated with the corresponding effects of MAb 32 and MAb 47 on the cytotoxic effects of $TNF_{\alpha}$ on cultured WEHI-164 tumour cells (Figure 2a). The activity of MAb 47 was found to be typical of 'inhibitory' antibodies; that is, the inhibitory activity was consistently observed in both the endothelial and tumour cell assays. Examination of the activation of cultured endothelial cells by $TNF_{\alpha}$ in the presence of MAbs 32 and 47 is shown in Figure 2b. Both antibodies significantly inhibited the $TNF_{\alpha}$-induced production of procoagulant as determined in the single stage clotting assay ($P<0.01$). Treatment of endothelial cells with MAbs 32 and 47 in the absence of $TNF_{\alpha}$ failed to induce procoagulant activity and therefore had no effect on the clotting time in the single stage clotting assay (data not shown). Similarly, neither $TNF_{\alpha}$ nor the antibodies themselves were cytotoxic to the endothelial cells under the conditions employed; however, $TNF_{\alpha}$ clearly stimulated the induction of procoagulant at these doses.

**Figure 1** Binding of $^{125}$I-$TNF_{\alpha}$ to WEHI-164 tumour cells and bovine aortic endothelial cells. (MAb 32, ■; MAb 47, □; Control MAb ○). Results are the mean±s.e.m. of triplicate determinations.

*Enhancement of $TNF_{\alpha}$-induced tumour regression of MAb 32*

$TNF_{\alpha}$, at a dose of 10 µg per injection, co-administered with a control MAb daily for 4 days to WEHI-164 tumour-bearing mice caused 50% reduction in tumour size (Figure 3). The degree of tumour regression observed was dose related such that animals treated with only 1 µg of $TNF_{\alpha}$ per day failed to show any reduction in tumour size. The tumour regressive activity of $TNF_{\alpha}$ was completely inhibited by pre-complexing the cytokine with anti-$TNF_{\alpha}$ MAb 47 before injection. In contrast, significant enhancement of tumour regression ($P<0.01$) was observed in mice treated with 10 µg $TNF_{\alpha}$ in complex with MAb 32 when compared to control mice (receiving 10 µg $TNF_{\alpha}$ in the presence of a control MAb). Enhanced tumour regression was also observed in mice treated with lower doses of $TNF_{\alpha}$ (1 µg) in the presence of MAb 32; indeed, at this dose of $TNF_{\alpha}$ in complex with MAb 32, an equivalent degree of regression was only observed following the treatment of mice with 10 µg of non-

**Figure 2** Effect of MAb 32 on $TNF_\alpha$-mediated tumour cell killing *in vitro* with cultured WEHI-164 fibrosarcoma cells **a**, and induction of endothelial cell procoagulant activity **b**. (MAb 32, ■; MAb 47, □; Control MAb ○). Results are the mean±s.e.m. of quadruplicate determinations.

**Figure 3** Enhancement of $TNF_\alpha$-mediated regression of WEHI-164 tumours *in vivo* by monoclonal antibody. MAb 32, ■; MAb 47, *; Control MAb, □. Mice were treated daily with $TNF_\alpha$ either pre-complexed with MAb 32 (50 μg) or after mixing with control MAb (50 μg). Tumour size was determined daily during the course of the experiment. The results show the mean±s.d. % change in tumour area at the completion of treatment (day 4). Differences observed between control MAb-$TNF_\alpha$ and MAb 32-$TNF_\alpha$ treated groups are significant ($P < 0.01$, unpaired *t*-test).

complexed $TNF_\alpha$ (i.e. with control antibody). At the 0.1 μg dose of $TNF_\alpha$ there was no apparent beneficial enhancement of tumour regression. Enhancement of $TNF_\alpha$-induced tumour regression by MAb 32 was also observed following the treatment of Meth A solid tumours *in vivo* (Figure 4). In contrast, however, the treatment of a Meth A tumour subline grown as ascites failed to give the observed enhancement response (data not shown).

FAb' fragments of MAb 32, prepared by papain digestion and purified on Protein A Sepharose as described in the Materials and methods, were found to enhance $TNF_\alpha$ induced tumour regression to the same degree as intact, bivalent MAb 32 (Figure 5). Neither intact MAb 32 nor FAb' MAb 32 caused tumour regression in the absence of $TNF_\alpha$.

### Discussion

The exploitation of genetic engineering technology has provided many protein hormones and mediators which may have clinical application in man; however, it is becoming progressively apparent that particular immunologically active recombinant molecules (e.g. $TNF_\alpha$, IL-1, IL-2, γ-IFN etc) retain high levels of toxicity *in vivo*. Reduction of the level of toxicity of such molecules may be a prerequisite for their more general therapeutic use. Since the toxicity of $TNF_\alpha$ may manifest as a direct result of its interaction with a variety of receptors on different tissues following systemic administration, we have examined the possibility of 'restricting' the specificity of this cytokine to particular receptor subsets with monoclonal antibodies (MAb). By employing this approach it is shown that the binding of $TNF_\alpha$ to different receptors can be selectively modulated by a particular MAb (MAb 32). These findings suggest that different regions of the cytokine are associated with its binding with different receptors and may account for the significant antibody-mediated enhancement of its activity described here.

Out of a panel of 13 monoclonal antibodies (MAbs) defining at least six distinct antigenic regions on $TNF_\alpha$, one specificity (MAb 32) has been identified which permits binding of the cytokine to tumour cell receptors (WEHI-164 cells) but not to sites on bovine aortic endothelial cells. The specificity of this MAb is unusual in that other inhibitory antibodies were characteristically found to block both the binding of $^{125}I$-$TNF_\alpha$ to WEHI-164 tumour cells and to bovine endothelial cells (see typically MAb 47). In view of the unique nature of MAb-antigen interactions (i.e. binding to a single site), interpretation of the distinct binding charac-

**Figure 4** Enhancement of $TNF_\alpha$-mediated regression of Meth A tumours *in vivo* by monoclonal antibody. MAb 32, ■; Control MAb, □. Mice were treated daily with $TNF_\alpha$ either pre-complexed with MAb 32 (50 μg) or after mixing with control MAb (50 μg). Tumour size was determined daily during the course of the experiment. The results show the mean±s.d. % change in tumour area at the completion of treatment (day 4). Differences observed between control MAb-$TNF_\alpha$ and MAb 32-$TNF_\alpha$ treated groups are significant ($P < 0.01$, unpaired *t*-test).

**Figure 5** Enhancement of tumour regressive activity of $TNF_\alpha$ by univalent FAb'-fragments of MAb 32. WEHI-164 tumour-bearing mice received a single dose of $TNF_\alpha$ (10 µg) mixed with one of the following: control MAb, MAb 32 or FAb' fragments from antibody MAb 32 or with PBS alone. Antibodies (50 µg) were pre-mixed with $TNF_\alpha$ 1 h prior to injection. One group of animals also received MAb 32 in the absence of $TNF_\alpha$.

teristics of $TNF_\alpha$-MAb 32 complexes to tumour cell and endothelial cell receptors indicates that the specificity of these respective binding sites may be different. Indeed, the existence of more than one $TNF_\alpha$ receptor has recently been documented (Hohman *et al.*, 1989; Brockhaus *et al.*, 1990). The selectivity of MAb 32 in inhibiting the binding of $TNF_\alpha$ to endothelial cells but not to tumour cells was confirmed by the demonstration that similar effects were observed in the corresponding bioassays with these cells in culture. Unlike MAb 32, the inhibitory antibody MAb 47 effectively blocked both the cytotoxicity of $TNF_\alpha$ on tumour cells and the ability of the cytokine to induce procoagulant in endothelial cell cultures.

The actions of $TNF_\alpha$ on cultured endothelial cells include activation of the pathways leading to the *de novo* induction of cell surface tissue factor and the loss of cell surface thrombomodulin (Nawroth & Stern, 1986). The possible *in vivo* consequences of these effects include the generation of thrombin and initiation of coagulation as well as decreases in activated protein C anti-coagulant activity. The recent demonstration that anti-tissue factor antibodies or protein C administration can abrogate some of the *in vivo* biological activities of $TNF_\alpha$ (toxic shock) lends support to this view (Nawroth & Stern, 1986; Taylor *et al.*, 1987; Edgington *et al.*, 1989). In contrast to the effects of MAb 32 on $TNF_\alpha$ activity in WEHI-164 cells, the antibody has been shown to be significantly more inhibitory than MAb 47 on the induction of procoagulant activity in cultured endothelial cells. The greater inhibitory potency of MAb 32 in the procoagulant assay, as compared with MAb 47 in receptor binding, is still unclear; however, it has been shown (Brett *et al.*, 1989) that endothelial cells may have more than one class of $TNF_\alpha$ receptor, of which the functionally representative one is reflected in the bioassay rather than in the radioreceptor assay (see below).

Predictably, the systemic administration of $TNF_\alpha$-MAb 32 complex would be anticipated to result in selective enhancement of the tumour regressive activity to the cytokine and possibly in inhibition of its effects on the endothelium. Here it has been shown that the pre-complexing of $TNF_\alpha$ with MAb 32 results in a significant (up to 10-fold) enhancement of the tumour regressive activity of the cytokine with no apparent concommitant increase in TNF toxicity. In contrast, other antibody specificities (inhibitory or otherwise) failed to produce this unique effect. The ability of univalent FAb' fragments, derived from MAb 32, to also enhance the cytotoxicity of $TNF_\alpha$ in this fashion indicates that the enhancement phenomenon is independent of antibody bivalency or Fc-region mediated effects (Aston *et al.*, 1989).

It is shown here that certain $TNF_\alpha$-MAb complexes can selectively bind to particular receptor subtypes *in vitro*. The phenomenon is not idiosyncratic to the concentrations of antibody or cytokine employed, as at high doses of antibody there is clear segregation of biological activity or binding to receptors. Similarly, the respective affinities of MAb 32 and MAb 47 (8.77 and $1.88 \times 10^9$ mmol $l^{-1}$) are unlikely to account for these observations. Other MAbs to $TNF_\alpha$ of analogous affinity to MAb 32 also failed to segregate the specificity of the cytokine in this manner. The distinct effects of MAb 32 on $TNF_\alpha$ activity may account, at least in part, for the substantial enhancement of anti-tumour activity of the cytokine against WEHI 164 and Meth A solid tumours *in vivo*. That is, when in complex with the cytokine, MAb 32 prevents the binding of $TNF_\alpha$ to the large numbers of receptors represented on the endothelium, whilst at the same time permitting binding and regression of the tumour itself. Therefore, in order for this type of hormonal enhancement to manifest, we propose that the complex must have access to at least two receptor subtype specificities *in vivo*: in such an event, 'restriction' of the mediator to a particular subtype by the antibody may occur. This mechanism would also account for the absence of any observed enhancement of cytotoxicity of $TNF_\alpha$ by MAb 32 on WEHI-164 cells *in vitro* and the observed lack of enhancement in the Meth A ascites tumour model. Previous cases of enhancement of hormonal activity by specific antisera were primarily attributed to antibody bivalency or prolongation of hormone half-life in the circulation (reviewed in Aston *et al.*, 1989). The potential benefits to the patient of circulating autoantibody to cytokines, particularly in relation to the latter mechanism, have also been discussed recently (Bendtzen *et al.*, 1990). It has been proposed in these studies that cytokine autoantibody occurs with high frequency in patients on cytokine therapy and may, in cases, benefit the patient by acting as a specific 'carrier' molecule. The recent identification of a natural $TNF_\alpha$ binding protein (Engelmann *et al.*, 1989), which may have regulatory effects on the cytokine *in vivo* and the existence of at least two structurally distinct receptors (Hohman *et al.*, 1989; Engelmann *et al.*, 1989; Loetscher *et al.*, 1990; Espevik *et al.*, 1990), would also lend support to the above hypothesis. Endothelial cells appear to express two TNF receptors which mediate different biological effects of TNF (Brett *et al.*, 1989). It appears that expression of tissue factor in response to TNF is signalled by a non-G protein linked receptor while increased vascular permeability in response to TNF occurs via a G protein-linked receptor. More recent evidence (Tartaglia *et al.*, 1991) has further indicated that the two TNF receptors initiate distinct signalling pathways that result in the induction of different cellular responses namely thymocyte proliferation and LM cytotoxicity. The antibody described here may not only enable further clinical trials with $TNF_\alpha$ but may have application in cancers where circulating $TNF_\alpha$ levels are high and evidence of coagulopathy is apparent.

We are grateful to J. Edwards, C. Livermore and J. McCormack for their expert technical assistance. We thank Dr Ian Clark and Dr Bill Cowden for initiating our interest in $TNF_\alpha$ and for their continued interest in the work.

### References

ASTON, R., COWDEN, W.B. & ADA, G.L. (1989). Antibody-mediated enhancement of hormone activity. *Molec. Immunol.*, **26**, 435.

ASTON, R., COOPER, L., HOLDER, A.T., IVANYI, J. & PREECE, M.A. (1985). Monoclonal antibodies to human growth hormone can distinguish between pituitary and genetically engineered forms. *Molec. Immunol.*, **22**, 271.

BENDTZEN, K., SVENSON, M., JONSSON, V. & HIPPE, E. (1990). Autoantibodies to cytokines-friends or foes? *Immunol. Today*, **11**, 167.

BEVILACQUA, M.P., POBER, J.S., MAJEAU, G.R., FIERS, W., COTRAN, R.S. & GIMBRONE, M.A. (1986). Recombinant tumor necrosis factor induces procoagulant activity in cultured human vascular endothelium: characteristation and comparison with the actions of interleukin 1. *Proc. Natl Acad. Sci. USA*, **83**, 4533.

BRETT, J., GERLACH, H., NAWROTH, P., STEINBERG, S., GODMAN, G. & STERN, D. (1989). Tumor necrosis factor/cachectin increases permeability of endothelial cell monolayers by a mechanism involving regulatory G proteins. *J. Exp. Med.*, **169**, 1977.

BROCKHAUS, M., SCHOENFELD, H.-J., SCHLAEGER, E., HUNZIHER, W., LESSLAUER, W. & LOETSCHER, H. (1990). Identification of two types of tumor necrosis factor receptors on human cell lines by monoclonal antibodies. *Proc. Natl Acad. Sci.*, **87**, 3127.

CARSWELL, E.A., OLD, L.J., KASSEL, R.L., GREEN, S., FIORE, N. & WILLIAMSON, B. (1975). An endotoxin-induced serum factor that causes necrosis of tumors. *Proc. Natl Acad. Sci. USA*, **72**, 3666.

CREAVEN, P.J., PLAGER, J.E., DUPERE, S. & 4 others (1987). Phase 1 clinical trial of recombinant human tumour necrosis factor. *Cancer Chemother. Pharmacol.*, **20**, 137.

EDGINGTON, T.S., MACKMAN, N., GREGORY, S.A. & MORRISSEY, J.H. (1989). Pleiotropic biologic effects *in vitro*: interaction with other cytokines. *2nd International Conference on Tumor Necrosis Factor and Related Cytokines*, Napa, California.

ENGLEMANN, H., ADERKA, D., RUBINSTEIN, M., ROTMANN, D. & WALLACH, D.J. (1989). A tumour necrosis factor-binding protein purified to homogeneity from human urine protects cells from tumour necrosis factor toxicity. *J. Biol. Chem.*, **264**, 11974.

ESPEVIK, T., BROCKHAUS, M., LOETSCHER, H., NONSTAD, U. & SHALABY, R. (1990). Characterization of binding and biological effects of monoclonal antibodies against a human tumor necrosis factor receptors. *J. Exp. Med.*, **171**, 415.

ESPEVICK, T. & NISSEN-MYER, J. (1986). A highly sensitive cell line, WEHI-164 clone 13, for measuring cytoxic factor/tumor necrosis factor from human monocytes. *J. Immunol. Methods*, **95**, 99.

GREEN, S., DOBRAJANSKY, A., CHIASSON, M.A., CARSWELL, E., SCHWARTZ, M.K. & OLD, L.J. (1977). *Corynebacterium parvum* as the priming agent in the production of tumour necrosis factor in the mouse. *J. Natl Cancer Inst.*, **59**, 1519.

HELSON, L., GREEN, F., CARSWELL, E. & OLD, L.J. (1975). Effect of tumour necrosis factor on cultured human melanoma cells. *Nature*, **258**, 731.

HOHMAN, H.-P., REMY, R., BROCKHAUS, M. & VAN LOON, A.G.M. (1989). Monoclonal antibodies against the TNF-receptor inhibit and down-regulate TNF binding. *J. Biol. Chem.*, **264**, 14927.

KIMURA, K., TAGUCHI, T., URUSHIZAKI, I. & 7 others (1987). Phase 1 study of recombinant human tumour necrosis factor. *Cancer Chemother. Pharmacol.*, **20**, 223.

LOETSCHER, H., PAN, Y.-C.-E., LAHM, H.-W. & 4 others (1990). Molecular cloning and expression of the 55 kd tumour necrosis factor receptor. *Cell*, **61**, 351.

NAWROTH, P.P. & STERN, D.M. (1986). Modulation of endothelial cell hemostatic properties by tumor necrosis factor. *J. Exp. Med.*, **163**, 740.

RATHJEN, D.A., COWAN, K., FURPHY, L.J. & ASTON, R. (1991). Antigenic structure of human tumour necrosis factor: recognition of distinct regions of TNFα by different tumour cell receptors. *Molec. Immunol.*, **28**, 79.

SELBY, P., HOBBS, S. VINER, C. & 7 others (1987). Tumour necrosis factor in man: clinical and biological observations. *Br. J. Cancer*, **56**, 803.

TARTAGLIA, L.A., WEBER, R.F., FIGARI, I.S., REYNOLDS, C., PALLADINO, M.A. & GOEDDEL, D.V. (1991). The two different receptors for tumour necrosis factor mediate distinct cellular responses. *Proc. Natl Acad. Sci. USA*, **88**, 9292.

TAYLOR, F.B., CHANG, A., ESMON, C.T. & 4 others (1987). Protein C prevents the coagulopathic and lethal effects of *Escherichia coli* infusion in the baboon. *J. Clin. Invest.*, **79**, 918.


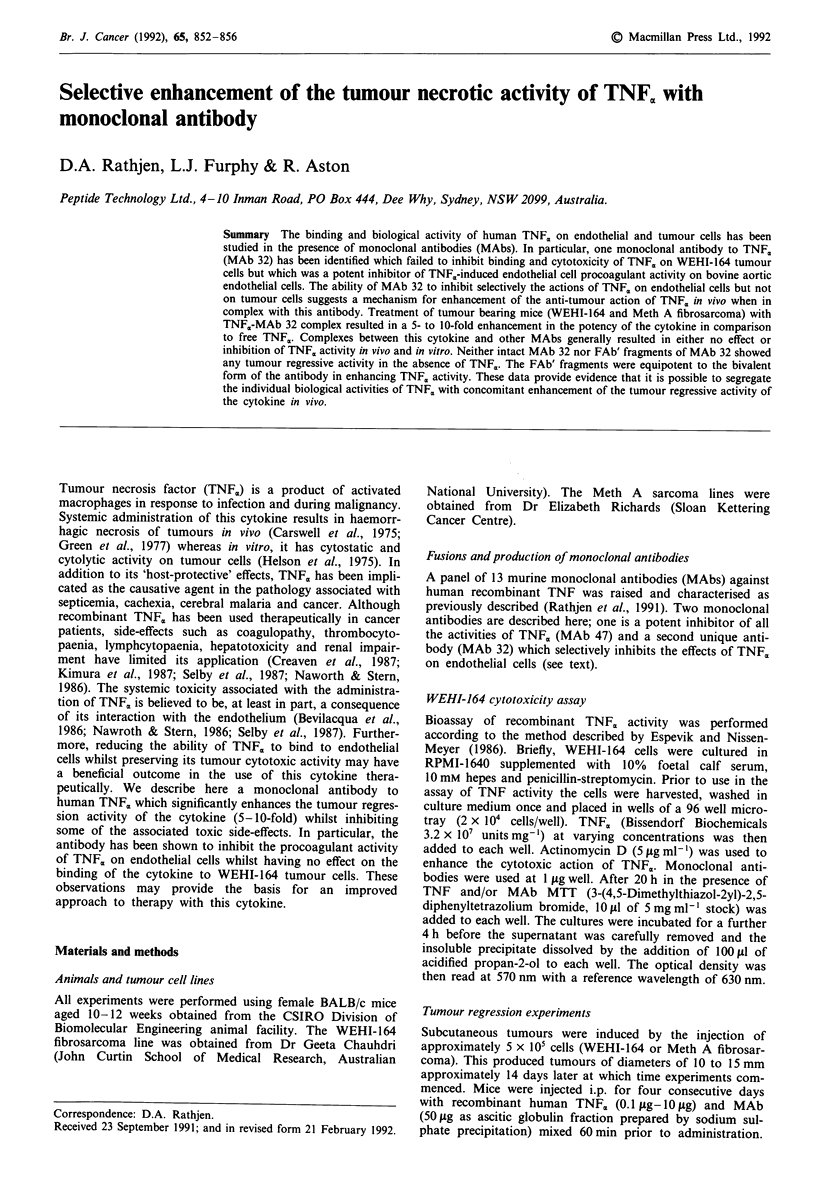

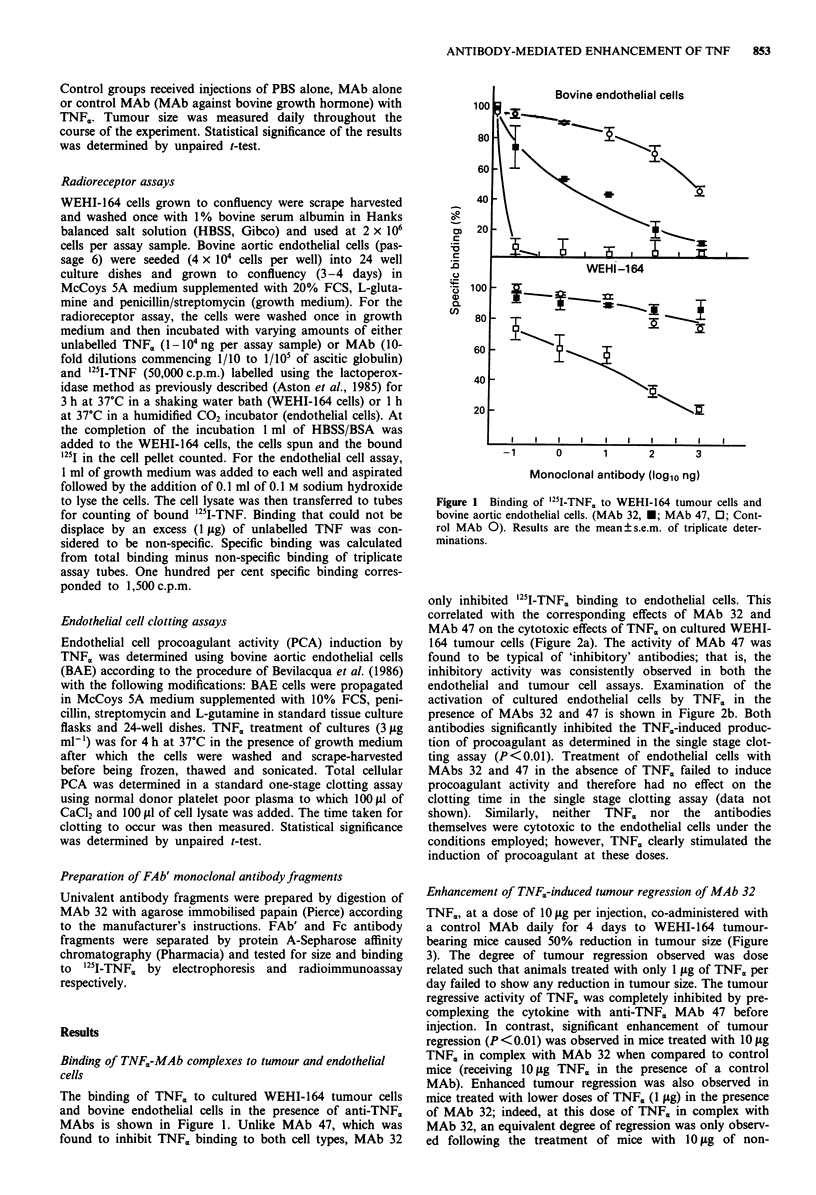

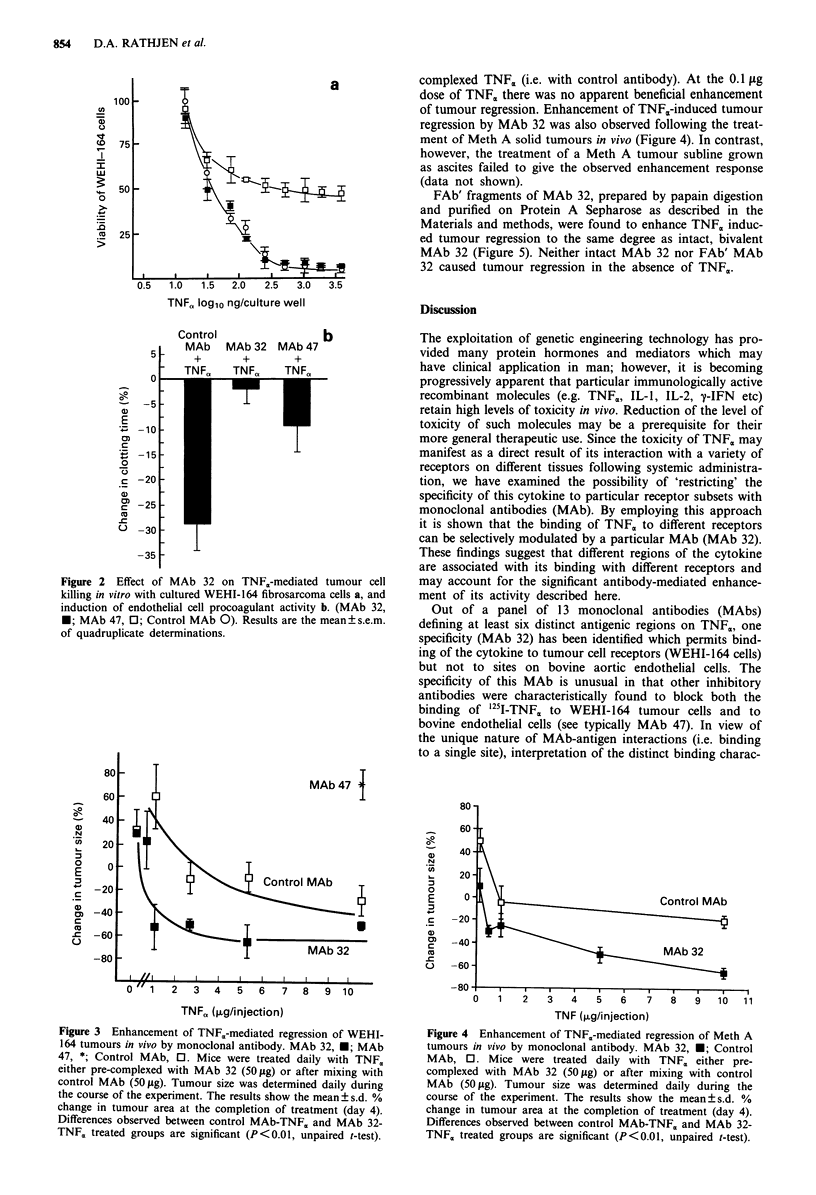

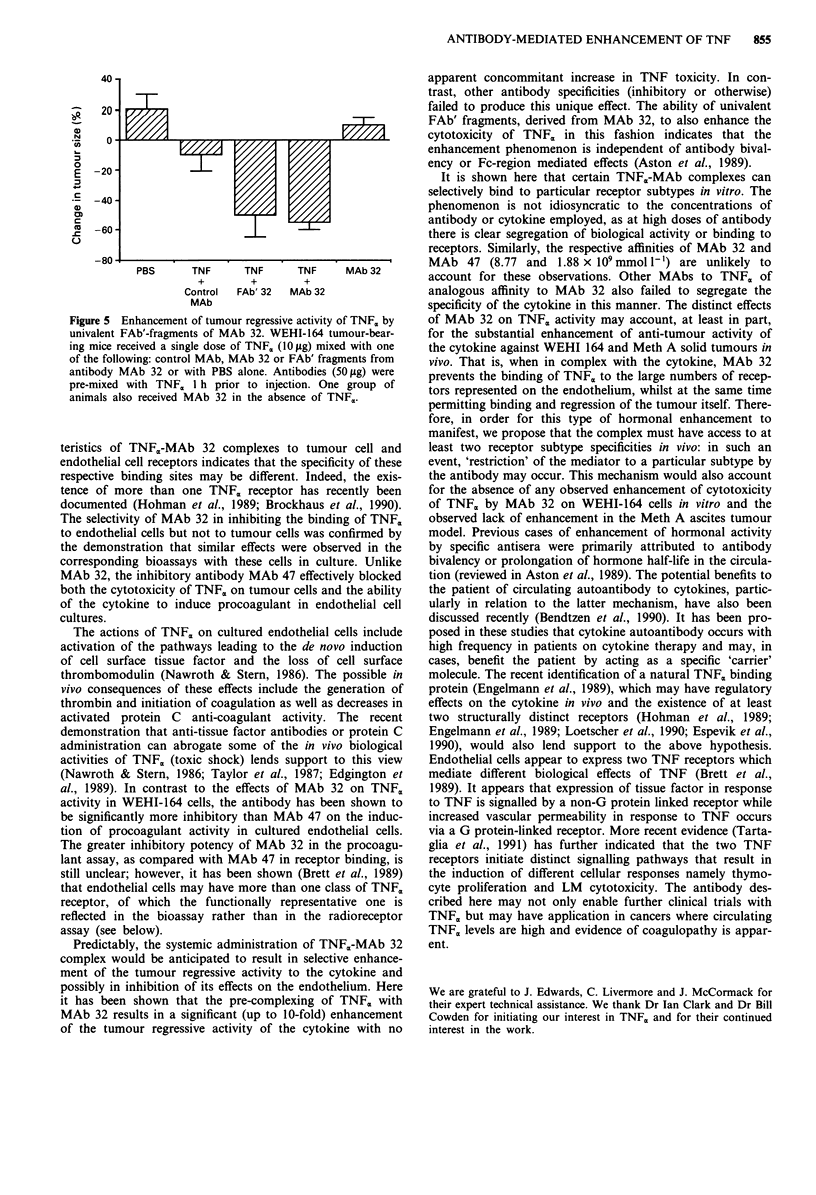

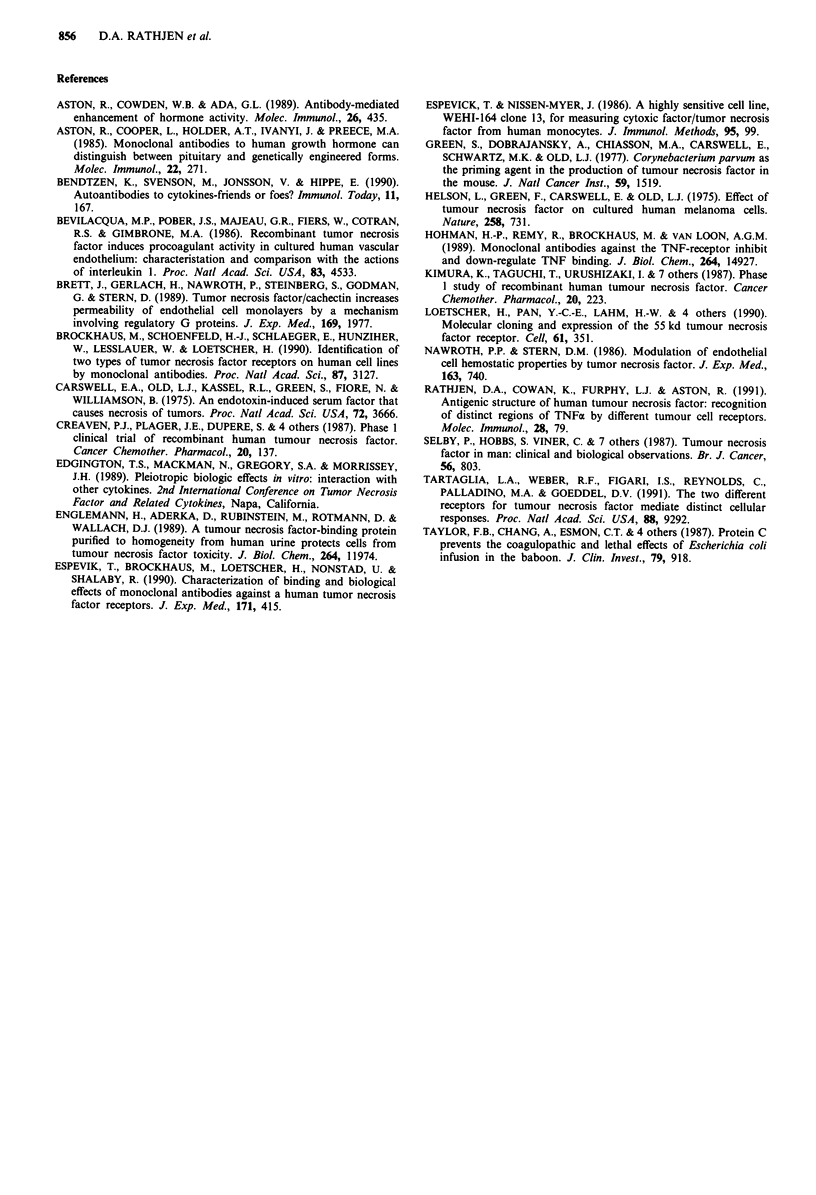


## References

[OCR_00600] Aston R., Cooper L., Holder A., Ivanyi J., Preece M. (1985). Monoclonal antibodies to human growth hormone can distinguish between pituitary and genetically engineered forms.. Mol Immunol.

[OCR_00596] Aston R., Cowden W. B., Ada G. L. (1989). Antibody-mediated enhancement of hormone activity.. Mol Immunol.

[OCR_00606] Bendtzen K., Svenson M., Jønsson V., Hippe E. (1990). Autoantibodies to cytokines--friends or foes?. Immunol Today.

[OCR_00611] Bevilacqua M. P., Pober J. S., Majeau G. R., Fiers W., Cotran R. S., Gimbrone M. A. (1986). Recombinant tumor necrosis factor induces procoagulant activity in cultured human vascular endothelium: characterization and comparison with the actions of interleukin 1.. Proc Natl Acad Sci U S A.

[OCR_00618] Brett J., Gerlach H., Nawroth P., Steinberg S., Godman G., Stern D. (1989). Tumor necrosis factor/cachectin increases permeability of endothelial cell monolayers by a mechanism involving regulatory G proteins.. J Exp Med.

[OCR_00624] Brockhaus M., Schoenfeld H. J., Schlaeger E. J., Hunziker W., Lesslauer W., Loetscher H. (1990). Identification of two types of tumor necrosis factor receptors on human cell lines by monoclonal antibodies.. Proc Natl Acad Sci U S A.

[OCR_00630] Carswell E. A., Old L. J., Kassel R. L., Green S., Fiore N., Williamson B. (1975). An endotoxin-induced serum factor that causes necrosis of tumors.. Proc Natl Acad Sci U S A.

[OCR_00634] Creaven P. J., Plager J. E., Dupere S., Huben R. P., Takita H., Mittelman A., Proefrock A. (1987). Phase I clinical trial of recombinant human tumor necrosis factor.. Cancer Chemother Pharmacol.

[OCR_00645] Engelmann H., Aderka D., Rubinstein M., Rotman D., Wallach D. (1989). A tumor necrosis factor-binding protein purified to homogeneity from human urine protects cells from tumor necrosis factor toxicity.. J Biol Chem.

[OCR_00651] Espevik T., Brockhaus M., Loetscher H., Nonstad U., Shalaby R. (1990). Characterization of binding and biological effects of monoclonal antibodies against a human tumor necrosis factor receptor.. J Exp Med.

[OCR_00657] Espevik T., Nissen-Meyer J. (1986). A highly sensitive cell line, WEHI 164 clone 13, for measuring cytotoxic factor/tumor necrosis factor from human monocytes.. J Immunol Methods.

[OCR_00662] Green S., Dobrjansky A., Chiasson M. A., Carswell E., Schwartz M. K., Old L. J. (1977). Corynebacterium parvum as the priming agent in the production of tumor necrosis factor in the mouse.. J Natl Cancer Inst.

[OCR_00668] Helson L., Green S., Carswell E., Old L. J. (1975). Effect of tumour necrosis factor on cultured human melanoma cells.. Nature.

[OCR_00673] Hohmann H. P., Remy R., Brockhaus M., van Loon A. P. (1989). Two different cell types have different major receptors for human tumor necrosis factor (TNF alpha).. J Biol Chem.

[OCR_00678] Kimura K., Taguchi T., Urushizaki I., Ohno R., Abe O., Furue H., Hattori T., Ichihashi H., Inoguchi K., Majima H. (1987). Phase I study of recombinant human tumor necrosis factor.. Cancer Chemother Pharmacol.

[OCR_00683] Loetscher H., Pan Y. C., Lahm H. W., Gentz R., Brockhaus M., Tabuchi H., Lesslauer W. (1990). Molecular cloning and expression of the human 55 kd tumor necrosis factor receptor.. Cell.

[OCR_00688] Nawroth P. P., Stern D. M. (1986). Modulation of endothelial cell hemostatic properties by tumor necrosis factor.. J Exp Med.

[OCR_00693] Rathjen D. A., Cowan K., Furphy L. J., Aston R. (1991). Antigenic structure of human tumour necrosis factor: recognition of distinct regions of TNF alpha by different tumour cell receptors.. Mol Immunol.

[OCR_00699] Selby P., Hobbs S., Viner C., Jackson E., Jones A., Newell D., Calvert A. H., McElwain T., Fearon K., Humphreys J. (1987). Tumour necrosis factor in man: clinical and biological observations.. Br J Cancer.

[OCR_00704] Tartaglia L. A., Weber R. F., Figari I. S., Reynolds C., Palladino M. A., Goeddel D. V. (1991). The two different receptors for tumor necrosis factor mediate distinct cellular responses.. Proc Natl Acad Sci U S A.

[OCR_00710] Taylor F. B., Chang A., Esmon C. T., D'Angelo A., Vigano-D'Angelo S., Blick K. E. (1987). Protein C prevents the coagulopathic and lethal effects of Escherichia coli infusion in the baboon.. J Clin Invest.

